# Evidence of the role of tick subolesin in gene expression

**DOI:** 10.1186/1471-2164-9-372

**Published:** 2008-08-02

**Authors:** José de la Fuente, Christine Maritz-Olivier, Victoria Naranjo, Patricia Ayoubi, Ard M Nijhof, Consuelo Almazán, Mario Canales, José M Pérez de la Lastra, Ruth C Galindo, Edmour F Blouin, Christian Gortazar, Frans Jongejan, Katherine M Kocan

**Affiliations:** 1 Department of Veterinary Pathobiology, Center for Veterinary Health Sciences, Oklahoma State University, Stillwater, OK 74078, USA; 2 Instituto de Investigación en Recursos Cinegéticos IREC (CSIC-UCLM-JCCM), Ronda de Toledo s/n, 13071 Ciudad Real, Spain; 3 Department of Biochemistry, Faculty of Natural and Agricultural Sciences, Agriculture Building, Lunnon Road, University of Pretoria, Pretoria, 0002, South Africa; 4 Department of Biochemistry and Molecular Biology, Oklahoma State University, Stillwater, OK 74078, USA; 5 Utrecht Centre for Tick-borne Diseases (UCTD), Department of Infectious Diseases and Immunology, Faculty of Veterinary Medicine, Utrecht University, Yalelaan 1, 3584CL, Utrecht, The Netherlands; 6 Facultad de Medicina Veterinaria y Zootecnia, Universidad Autónoma de Tamaulipas, Km. 5 carretera Victoria-Mante, CP 87000 Cd. Victoria, Tamaulipas, Mexico; 7 Department of Veterinary Tropical Diseases, Faculty of Veterinary Science, University of Pretoria, Private Bag X04, 0110, Onderstepoort, South Africa

## Abstract

**Background:**

Subolesin is an evolutionary conserved protein that was discovered recently in *Ixodes scapularis *as a tick protective antigen and has a role in tick blood digestion, reproduction and development. In other organisms, subolesin orthologs may be involved in the control of developmental processes. Because of the profound effect of subolesin knockdown in ticks and other organisms, we hypothesized that subolesin plays a role in gene expression, and therefore affects multiple cellular processes. The objective of this study was to provide evidence for the role of subolesin in gene expression.

**Results:**

Two subolesin-interacting proteins were identified and characterized by yeast two-hybrid screen, co-affinity purification and RNA interference (RNAi). The effect of subolesin knockdown on the tick gene expression pattern was characterized by microarray analysis and demonstrated that subolesin RNAi affects the expression of genes involved in multiple cellular pathways. The analysis of subolesin and interacting protein sequences identified regulatory motifs and predicted the presence of conserved protein kinase C (PKC) phosphorylation sites.

**Conclusion:**

Collectively, these results provide evidence that subolesin plays a role in gene expression in ticks.

## Background

Ticks are obligate hematophagous ectoparasites of wild and domestic animals and humans, and are important vectors of diseases to humans and animals worldwide [1]. Ticks are classified in the subclass Acari, order Parasitiformes, suborder Ixodida and are distributed worldwide from Arctic to tropical regions [2]. Despite efforts to control tick infestations, these ectoparasites remain a serious threat for human and animal health [3,4]. Recently, both vaccine studies using key tick antigens as well as characterization of tick gene function by RNA interference (RNAi) have provided new information on genes that impact tick life cycle and the tick-pathogen interface [3-6].

One of these genes, subolesin (also called 4D8), was discovered recently in *Ixodes scapularis *and was shown by both RNAi and immunization with recombinant proteins to protect against tick infestations, resulting in reduced tick survival, feeding and reproduction [7-11]. The silencing of subolesin expression by RNAi in ticks resulted in degeneration of gut, salivary gland, reproductive and embryonic tissues as well as causing sterility in males [10-13]. In addition, targeting subolesin by RNAi or vaccination decreased the ability of ticks to become infected with *Anaplasma marginale *or *A. phagocytophilum *[14,15].

Evidence of the conservation of subolesin throughout evolution was provided by the high homology of amino acid sequences in higher eukaryotes, which suggests an essential conserved biological function for this protein [10]. For example, the expression of subolesin orthologs has been detected in a variety of adult and immature tissues of several tick species [8,10,12], *Drosophila melanogaster *[16,17] and *Caenorhabditis elegans *[18]. These studies have suggested that subolesin orthologs may be involved in the control of developmental processes in these organisms [8,10,12,16-18]. However, despite the important role that subolesin plays in tick reproduction, development and pathogen infection, the biological function of subolesin and its orthologs has not been reported.

Because of the profound effect of subolesin knockdown in ticks and other organisms we hypothesized that subolesin may play a role in gene expression, thus affecting multiple cellular processes. Herein, gene expression is understood as the process by which a gene gets turned on in a cell to make RNA and proteins and therefore may be affected at the transcriptional and/or translational levels. The objective of this study was to provide evidence for the role of subolesin in gene expression through a combination of methodological approaches that included characterization of subolesin-interacting proteins, the effect of gene knockdown on tick gene expression pattern and the prediction of subolesin post-translational modifications. Although the biological function of subolesin is unkown, the results provided evidence that this protein plays a role in gene expression in ticks and most likely other organisms.

## Results

### Identification of subolesin interaction proteins by yeast two-hybrid screen and co-affinity purification

**Figure 1:**
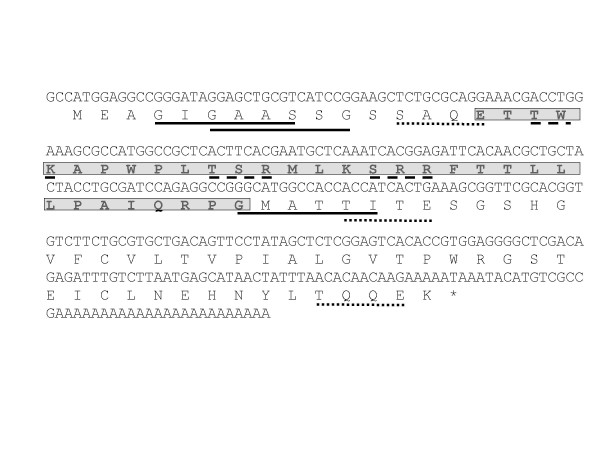
**Analysis of GI sequence encoding for subolesin-interacting protein.** GI nucleotide and deduced amino acid sequences are shown. The GI sequence contains a transduction/transcription domain (boxed letters) and multiple N-myristoylation (solid line) and CK2 (dotted line) and PKC (dashed line) phosphorylation sites.

**Figure 2:**
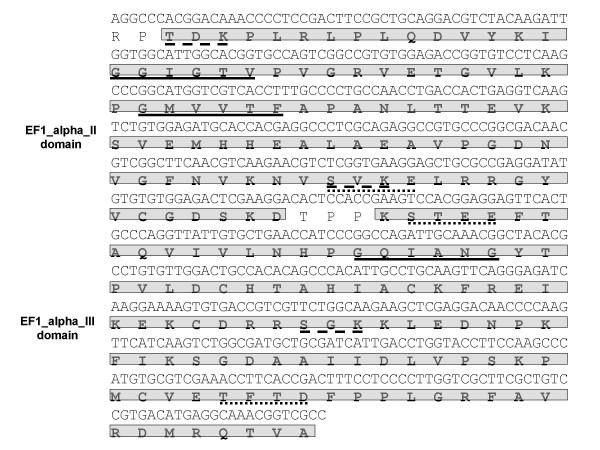
**Analysis of GII sequence encoding for subolesin-interacting protein.** GII nucleotide and deduced amino acid sequences are shown. The GII sequence contains EF1_alpha_II and EF1_alpha_III domains (boxed letters) and multiple N-myristoylation (solid line) and CK2 (dotted line) and PKC (dashed line) phosphorylation sites.

For discovery of subolesin-interacting proteins, subolesin was used as a bait to search for preys in *Rhipicephalus (Boophilus) microplus *using the yeast two-hybrid system. Two sequences, GI and GII, were identified that encoded for candidate subolesin-interacting proteins. These sequences were represented in 50% and 10% of the positive clones, respectively. BLAST analysis of the GI sequences did not result in identity to known sequences, except for the EST910636 from a *R. microplus *cDNA library. However, a transduction/transcription domain was found within the GI open reading frame (ORF) (Fig. 1). The GII sequence was 99% identical (Expect = 4e-102) to *Amblyomma *sp. elongation factor-1 alpha (EF-1a; [Genbank:AAK12647]) and to other proteins containing EF1_alpha_II and EF1_alpha_III domains (Fig. 2). Both GI and GII sequences contained multiple N-myristoylation and casein kinase II (CK2) and protein kinase C (PKC) phosphorylation sites (Figs. 1 and 2).

**Figure 3:**
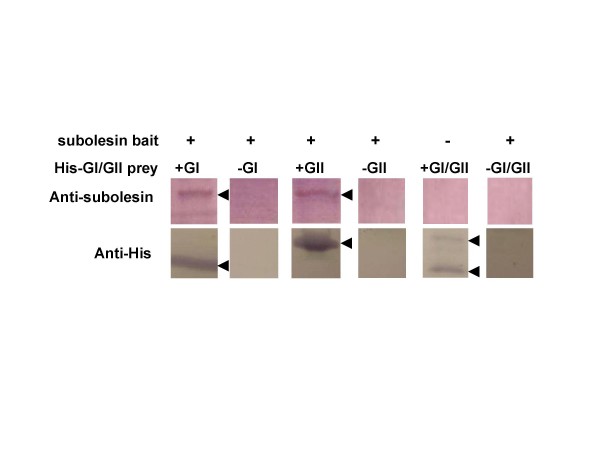
**Co-affinity purification assays.** The supernatant of GI- and GII-expressing *E. coli *cell lysates were loaded onto Ni-NTA columns, washed, then loaded with purified recombinant subolesin and washed again before protein elution for SDS-PAGE and Western blot. The top panel shows subolesin bait detection after affinity purification on Ni-NTA columns in the presence (+) or absence (-) of His-tagged GI/GII protein extracts, demonstrating binding to His-tagged prey. The bottom panel shows detection of GI/GII His-prey after affinity purification on Ni-NTA columns.

The proteins encoded by GI and GII were expressed in *Escherichia coli *fused to a 6xHis tag with molecular weights of 10 and 22 kDa, respectively and the interaction of these proteins with subolesin was confirmed by co-affinity purification on Ni-NTA columns (Fig. 3). In the absence of either GI or GII recombinant proteins or in the presence of an unrelated His-tagged protein, co-purification of subolesin did not occur, confirming the specificity of the interaction between subolesin and GI or GII (Fig. 3 and data not shown).

### Characterization of the effect of GI and GII knockdown on tick survival, feeding, oviposition, hatching and egg development

**Figure 4:**
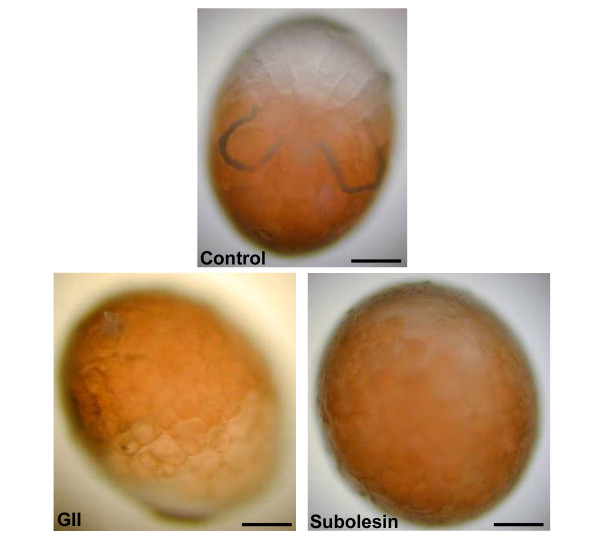
**Representative *R. microplus *eggs from replete female ticks injected with injection buffer alone (saline control) showing normal development, or GII and subolesin dsRNA showing an undifferentiated egg mass.** Eggs were incubated at 27°C and 95% relative humidity. Photographs were taken 22 dpi of replete females and 19 days after oviposition. Bar = 0.1 mm.

RNAi was used to study the effect of the knockdown of subolesin-interacting protein-coding genes, GI and GII, on female tick survival, feeding, oviposition, hatching and egg development. Subolesin, GI or GII dsRNAs or injection buffer (saline control) were injected into either unfed or replete *R. microplus *female ticks. The injection of GI dsRNA did not affect tick survival, feeding and oviposition, a fact that correlated with the absence of GI knockdown in ticks (Table 1). Although GI expression was reduced in tick eggs, it did not affect hatching (Table 2). Gene knockdown was demonstrated for GII and subolesin in both ticks and eggs (Tables 1 and 2). GII knockdown produced a phenotype similar to that obtained with the silencing of subolesin expression. The injection of GII dsRNA in unfed ticks resulted in reduced tick survival, feeding, oviposition and hatching when compared to control ticks (Table 1). When GII dsRNA was injected into replete ticks, hatching and egg development were affected (Table 2 and Fig. 4). Undifferentiated egg masses were observed in eggs oviposited by replete ticks injected with GII and subolesin dsRNA when compared to saline injected controls (Fig. 4).

**Table 1:** *R. microplus *tick survival, weight, oviposition and hatching after RNAi in unfed female ticks.

**Experimental group**	**Number of ticks**	**Expression silencing (%)^a^**	**Tick weight (mg)^b^**	**Mortality (%)^c^**	**Eggs per tick (mg)^d^**	**Hatching rate (%)**
Saline control	76	---	295 ± 85	21	111 ± 75	> 90
GI	60	-44 ± 29	266 ± 114	17	110 ± 72	> 90
GII	63	98 ± 0.4*	131 ± 161**	60***	1 ± 4****	0*****
Subolesin	66	91 ± 3*	73 ± 94**	80***	0****	ND

^a^The expression silencing of target genes was determined by real-time RT-PCR and average ± SD mRNA levels calculated and compared between dsRNA and saline-injected control ticks by Student's t-test with unequal variance (*P < 0.01). Amplification efficiencies were normalized against β-actin.

^b^Ticks which completed feeding and those removed after 30 days (15 days after saline or dsRNA injection) were weighed individually and average ± S.D. calculated and compared between dsRNA and saline-injected control ticks by Student's t-test with unequal variance (**P < 0.01).

^c^Tick mortality was evaluated as the ratio of dead female ticks after day 30 (15 days after saline or dsRNA injection) to the total number of female ticks placed on the animal and was compared between dsRNA and saline-injected control ticks by χ^2^-test (***α < 0.001).

^d^The egg mass oviposited by each tick was weighed individually and average ± S.D. calculated and compared between dsRNA and saline-injected control ticks by Student's t-test with unequal variance (****P < 0.01).

^e^The hatching rate was determined 6 weeks after oviposition and compared with the saline-injected control ticks by Student's t-test with unequal variance (*****P < 0.01).

Abbreviations: ND, not determined because ticks did not laid eggs.

**Table 2:** Weight, oviposition and hatching of replete *R. microplus *female ticks after RNAi.

**Experimental group**	**Tick weight (mg)^a^**	**Eggs per tick (mg)^b^**	**Hatching rate (%)^c^**	**Expression silencing (%)^d^**
Saline control	318 (272–367)	173 (128–229)	> 90	---
GI	318 (260–369)	123 (31–175)	> 90	55 ± 8**
GII	315 (267–349)	132 (95–182)	0.2*	84 ± 5**
Subolesin	318 (270–360)	142 (101–175)	0.4*	46 ± 8**

^a^Replete *R. microplus *ticks were collected and weighed individually before injection with dsRNA, all within 6 hours after dropping of the host. The average weight and variation (between parentheses) of each group are shown. No significant statistical differences were observed (P > 0.05).

^b^The egg mass oviposited by each tick was weighed individually. The average egg mass and variation (between parentheses) was calculated and compared with the saline-injected control ticks by Student's t-test with unequal variance. No significant statistical differences were observed (P > 0.05).

^c^The hatching rate was determined 6 weeks post oviposition and compared with the saline-injected control ticks by Student's t-test with unequal variance (*P < 0.01).

^d^The expression silencing of target genes was determined in eggs by real-time RT-PCR and average ± S.D. mRNA levels calculated and compared between dsRNA and saline-injected control ticks by Student's t-test with unequal variance (**P < 0.05). Amplification efficiencies were normalized against β-actin.

### Characterization of the effect of subolesin knockdown on tick gene expression profile

The results of the subolesin-protein interactions and RNAi experiments with GI and GII, encoding for subolesin-interacting proteins, suggested that subolesin may be involved in gene expression in ticks. Therefore, the subsequent experiments were directed towards analyzing the effect of subolesin knockdown on the tick gene expression profile.

**Figure 5:**
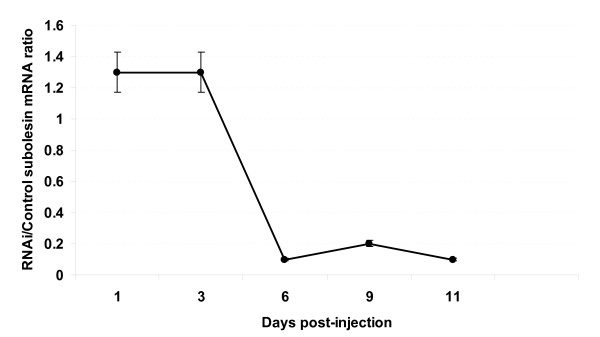
**Kinetics of subolesin RNAi.** Ticks were injected with subolesin dsRNA (RNAi group) or injection buffer alone (control group). Three ticks from each group were collected at 1, 3, 6, 9 and 11 days post injection to determine mean ± SD subolesin mRNA ratio by real-time RT-PCR.

In the first experiment, the kinetics of subolesin silencing by RNAi was investigated in unfed *I. scapularis *female ticks. The results showed that over 90% silencing of subolesin transcription was obtained 6 days post injection (dpi) and continued until at least 11 dpi (Fig. 5). Significant differences (P = 0.02) in tick weight between subolesin dsRNA (78 ± 33 mg) and saline injected controls (142 ± 47 mg) were observed in ticks that completed feeding on the host in the presence of males at 11 dpi.

In the second experiment, unfed *I. scapularis *female ticks were injected with subolesin dsRNA or injection buffer alone. Ticks were then placed on a sheep without males and collected at 6 and 9 dpi, by which time subolesin knockdown was regarded as significant based on the RNAi kinetics experiment (Fig. 5). Subolesin knockdown was corroborated by real-time RT-PCR as 81 ± 3% and 79 ± 5% silencing at 6 and 9 dpi, respectively. A group of ticks collected after 10 days of feeding with males was used to corroborate the effect of subolesin knockdown on tick weight, giving results similar to those obtained in the RNAi kinetics experiment described above. The ticks collected at 6 and 9 dpi were then dissected and used to extract RNA for suppression-subtractive hybridization (SSH) and microarray construction and analysis.

**Figure 6:**
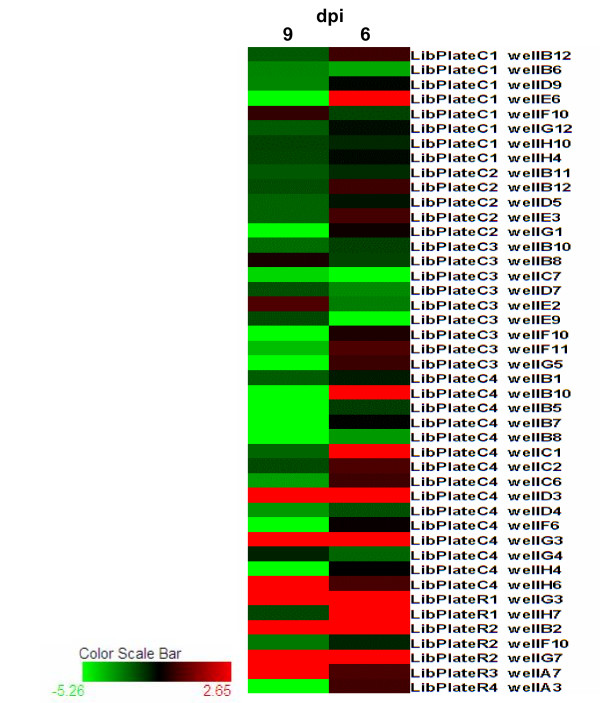
**Effect of subolesin knockdown on tick gene expression pattern.** The expression fold change was determined by microarray hybridization at 6 and 9 days post injection (dpi). Clone ID (SSH library plate and well) are shown and correspond to entries in Table 3. The graph was constructed with the HCE software .

The SSH libraries constructed with the RNA of subolesin dsRNA and saline injected ticks collected at 9 dpi were used for random clone amplification and microarray construction. This microarray was enriched for genes differentially expressed after subolesin knockdown. The microarray was hybridized with total RNA prepared from subolesin dsRNA- and saline-injected ticks at 6 and 9 dpi to evaluate how the expression pattern of candidate differentially expressed genes varied at two different time points after subolesin knockdown (Table 3). The results revealed 34 unique (not redundant) genes that were down or up-regulated by at least two-fold after subolesin knockdown at 6 or 9 dpi (Fig. 6). Of these genes, 28 were down-regulated and 6 were up-regulated after subolesin RNAi. Of the down-regulated genes, 4 were down-reglated at 6 dpi, 20 at 9 dpi and 4 at both 6 and 9 dpi (Fig. 6). Up-regulated genes were found at 9 dpi only (Fig. 6). In the microarray analysis, subolesin expression was silenced by approximately 35% at both 6 and 9 dpi. Although 62% of the unique differentially expressed genes did not have homology to available sequences or were identical to genes with unknown function, the remaining sequences indicated that various biological processes were affected by subolesin knockdown. These included regulation of protein and nucleic acid metabolism (54% of protein ontology assignments), energy pathways (15%), immunity (15%), cell communication and signal transduction (8%) and transport (8%).

**Table 3:** *I. scapularis *differentially expressed genes after subolesin knockdown.

**Clone ID^a^**	**Accession number, name and species^b^**	**Fold change (6 dpi)^c^**	**SD (6 dpi)^d^**	**Fold change (9 dpi)^c^**	**SD (9 dpi)^d^**
LibPlateC1_wellB12	[Genbank:AAT92189] KUN-6 (*Ixodes pacificus*)	-1.1196	0.2519	-2.2057	0.3109
LibPlateC1_wellB6	[Genbank:AAZ29240] copper/zinc superoxide dismutase (*Macrobrachium rosenbergii*)	-2.7645	0.5455	-2.5136	0.2930
LibPlateC1_wellD9	[Genbank:AAY66901] histone 2B (*Ixodes scapularis*)	-1.5922	1.5332	-2.5298	0.9579
LibPlateC1_wellE6	[Genbank:AAY66498] putative secreted salivary WC peptide (*Ixodes scapularis*)	1.2320	1.4160	-5.2598	1.3686
LibPlateC1_wellF10	[Genbank:AAY66817] anticoagulant Salp9-like (*Ixodes scapularis*)	-2.0332	0.2959	-1.2037	0.1124
LibPlateC1_wellG12	[Genbank:AAZ29240] copper/zinc superoxide dismutase (*Macrobrachium rosenbergii*)	-1.6426	0.2527	-2.2118	0.4974
LibPlateC1_wellH10	No homology found	-1.8346	0.7216	-2.0350	1.0821
LibPlateC1_wellH4	No homology found	-1.6161	0.4640	-2.0602	0.5671
LibPlateC2_wellB11	[Genbank:AAY66498] putative secreted salivary WC peptide (*Ixodes scapularis*)	-1.8612	0.1952	-2.1981	0.1664
LibPlateC2_wellB12	No homology found	-1.1313	0.1588	-2.1133	0.2023
LibPlateC2_wellD5	No homology found	-1.6882	0.0965	-2.2689	0.2158
LibPlateC2_wellE3	[Genbank:AAY66498] putative secreted salivary WC peptide (*Ixodes scapularis*)	-1.0794	0.2033	-2.2673	0.2302
LibPlateC2_wellG1	[Genbank:AAM93633|AF483711_1] putative secreted protein (*Ixodes scapularis*)	-1.4409	0.2000	-3.5351	0.3250
LibPlateC3_wellB10	[Genbank:AAH67494] HIST1H3I protein (*Homo sapiens*)	-2.0111	0.2174	-2.3299	0.4323
LibPlateC3_wellB8	No homology found	-2.0438	1.5307	-1.3772	0.5537
LibPlateC3_wellC7	[Genbank:AAZ29240] copper/zinc superoxide dismutase (*Macrobrachium rosenbergii*)	-4.1699	0.1770	-3.1053	0.0638
LibPlateC3_wellD7	[Genbank:AAZ29240] copper/zinc superoxide dismutase (*Macrobrachium rosenbergii*)	-2.5487	0.1977	-2.1210	0.4374
LibPlateC3_wellE2	[Genbank:XP_624446.2] Predicted similar to ruby CG11427-PA isoform 2 (*Apis mellifera*)	-2.4606	1.6665	-1.0115	0.3122
LibPlateC3_wellE9	[Genbank:AAZ29240] copper/zinc superoxide dismutase (*Macrobrachium rosenbergii*)	-3.4248	0.3717	-2.0792	0.2988
LibPlateC3_wellF10	[Genbank:AAM93633|AF483711_1] putative secreted protein (*Ixodes scapularis*)	-1.3583	0.2449	-3.9731	0.1679
LibPlateC3_wellF11	[Genbank:AAM93633|AF483711_1] putative secreted protein (*Ixodes scapularis*)	-1.0215	0.1441	-2.9246	0.1172
LibPlateC3_wellG5	[Genbank:AAM93633|AF483711_1] putative secreted protein (*Ixodes scapularis*)	-1.1451	0.2311	-3.7711	0.1890
LibPlateC4_wellB1	No homology found	-1.7450	0.6400	-2.2268	0.3409
LibPlateC4_wellB10	[Genbank:AAQ01562] von Willebrand factor (*Ixodes ricinus*)	1.1202	0.3737	-3.9449	0.2800
LibPlateC4_wellB5	[Genbank:ABD83654] hemelipoglycoprotein precursor (*Dermacentor variabilis*)	-1.9958	0.5689	-3.5813	0.6757
LibPlateC4_wellB7	[Genbank:AAP84098] ML domain-containing protein (*Ixodes ricinus*)	-1.5703	0.3164	-4.5599	0.4105
LibPlateC4_wellB8	No homology found	-2.6551	0.3564	-3.5944	0.3325
LibPlateC4_wellC1	[Genbank:AAY66502] secreted salivary gland protein (*Ixodes scapularis*)	1.0009	0.2427	-2.2922	0.3518
LibPlateC4_wellC2	No homology found	-1.0286	0.0610	-2.0853	0.1338
LibPlateC4_wellC6	No homology found	-1.1217	0.1682	-2.7104	0.1235
LibPlateC4_wellD3	No homology found	1.6859	0.2075	2.6468	0.1954
LibPlateC4_wellD4	[Genbank:XP_974124] PREDICTED: similar to CG2972-PA (*Tribolium castaneum*)	-2.1335	0.2315	-2.6578	0.3642
LibPlateC4_wellF6	[Genbank:BAE53722] aspartic protease (*Haemaphysalis longicornis*)	-1.4881	0.2385	-4.8576	0.3690
LibPlateC4_wellG3	[Genbank:AAY66629] putative secreted salivary protein (*Ixodes scapularis*)	1.0601	0.1625	2.0049	0.1481
LibPlateC4_wellG4	[Genbank:XP_794044] Predicted similar to Endoplasmic reticulum-golgi intermediate	-2.2835	0.2324	-1.7916	0.5510
LibPlateC4_wellH4	[Genbank:AAY66660] putative salivary secreted protein (*Ixodes scapularis*)	-1.5502	0.2177	-5.1239	0.2056
LibPlateC4_wellH6	[Genbank:AAY66982] cyclophilin A (*Ixodes scapularis*)	-1.0545	0.0098	2.0388	0.2239
LibPlateR1_wellG3	No homology found	1.2792	0.2664	2.2118	0.1902
LibPlateR1_wellH7	[Genbank:AAY66764] putative secreted salivary protein (*Ixodes scapularis*)	1.2470	0.0447	-2.0598	0.0708
LibPlateR2_wellB2	[Genbank:AAY66713] putative secreted salivary protein (*Ixodes scapularis*)	1.4186	0.2490	2.2141	0.2145
LibPlateR2_wellF10	[Genbank:AAK97818|AF209915_1] 16 kDa salivary gland protein A (*Ixodes scapularis*)	-1.8100	0.4667	-2.4275	0.3690
LibPlateR2_wellG7	[Genbank:AAY66713] putative secreted salivary protein (*Ixodes scapularis*)	1.4101	0.1056	2.1495	0.1347
LibPlateR3_wellA7	No homology found	-1.0338	0.1570	2.0213	0.1464
LibPlateR4_wellA3	No homology found	-1.0990	1.1277	-3.4438	1.6338

^a^Clone ID identifies the clone based on SSH Library Plate_well.

^b^Name: This output only contains descriptions of the top blast hit (most significant alignment based on E value).

^c^Fold change is of the global normalized ratio (log2(635/532)) of background-corrected means averaged between replicates (determined from valid spots only). Only genes down-regulated (negative values) and up-regulated (positive values) after subolesin knockdown by at least two fold at 6 or 9 dpi were considered.

^d^SD is the standard deviation determined from the normalized average log2 ratio (determined from valid spots only).

Three of the down-regulated genes after subolesin knockdown, identical to *I. scapularis *putative secreted salivary WC peptide [Genbank:AAY66498] and putative secreted protein [Genbank:AAM93633] and *Macrobrachium rosenbergii *copper/zinc superoxide dismutase (Cu-Zn SOD) [Genbank:AAZ29240], were selected to corroborate the results of the microarray analysis by real-time RT-PCR. The results demonstrated that the expression of these genes was silenced after subolesin RNAi by 98% and 100% (for [Genbank:AAM93633]), 41% and 59% (for [Genbank:AAY66498]) and 93% and 74% (for [Genbank:AAZ29240]) at 6 and 9 dpi, respectively. Ticks injected with an unrelated 4A8 dsRNA control did not show silencing of these genes after RNAi. The possibility of subolesin RNAi off-target effects was analyzed and complementary 7 bp regions were not found between all possible 20–22 bp subolesin siRNAs and tick cDNA sequences of differentially expressed genes identified in the microarray analysis.

### Prediction of subolesin conserved post-translational modifications

**Figure 7:**
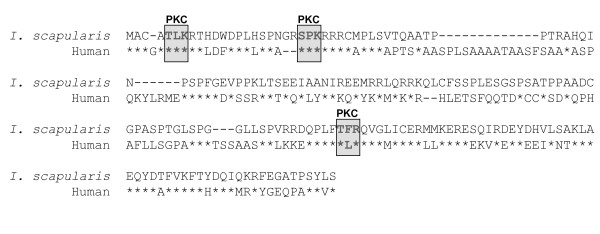
**Prediction of subolesin post-translational modifications.** Sequence alignment of *I. scapularis *subolesin [Genbank:AAV67031] and human ortholog [Genbank:NP_060534] proteins using the one letter amino acid code. Identical amino acids are indicated with asterisks. Three conserved PKC phosphorylation sites were predicted by PIR searching against the PROSITE database.

*I. scapularis *subolesin [Genbank:AAV67031] and human ortholog C6orf166 [Genbank:NP_060534] proteins were compared to predict conserved post-translational modifications. Three conserved PKC phosphorylation sites were found (Fig. 7), which were also present in all known tick subolesin protein sequences (data not shown).

## Discussion

Subolesin, discovered and characterized in *I. scapularis *as a tick protective antigen [7-9], is an evolutionary conserved protein which is involved in modulation of tick blood digestion, reproduction and development [10-13]. In other organisms, subolesin orthologs may be involved in the control of developmental processes [16-18]. Although the function of subolesin is unknown, these results suggest a conserved function for subolesin. Because of the profound effect of subolesin knockdown in ticks and other organisms [10-13,18], our hypothesis was that subolesin may have a role in gene expression, thus affecting multiple cellular processes. Therefore, the objective of this study was to provide evidence of the role of subolesin in gene expression. To test this hypothesis, three experiments were conducted. In the first series of experiments, subolesin-interacting proteins were identified and characterized in *R. microplus*, suggesting the interaction of subolesin with regulatory proteins. Therefore, in the second series of experiments, the effect of subolesin knockdown was analyzed in *I. scapularis *and showed the effect of subolesin on gene expression affecting different biological processes. Finally, post-translational modifications were predicted for tick subolesin. All together, the results of these experiments suggested a role for tick subolesin in gene expression.

To identify proteins that interact with subolesin in yeast two-hybrid experiments, we used a cDNA library obtained from tick eggs because subolesin is expressed in tick embryos and gene knockdown affects egg development [8,12]. Two genes, GI and GII, were identified encoding for proteins that interact with tick subolesin. These proteins contained domains and post-translational modification sites found in proteins with regulatory functions. The transduction/transcription domain found in GI is present in phosphorylated proteins involved in transcriptional regulation and other cell functions related to gene expression [19,20]. The EF1_alpha_II and EF1_alpha_III domains present in GII are found in proteins with different functions such as protein biosynthesis, DNA binding, transcriptional regulation, RNA processing, structural constituent of cytoskeleton as well as ATP and GTP binding that are also involved in gene expression (see for example proteins with accession numbers [Genbank:EAY95388, Genbank:XP_657651, Genbank:XP_404184, Genbank:XP_312333, Genbank:XP_001651261]).

The results reported herein suggested that subolesin may interact with regulatory proteins. In other organisms, subolesin orthologs interact with proteins with gene expression regulatory activities. The human subolesin ortholog interacts with LNXp80 [Genbank:AK056823], DIPA [Genbank:NM_006848] and SPG21 [Genbank:NM_016630] proteins. LNX is an E3 ubiquitin-protein ligase that mediates ubiquitination and subsequent proteasomal degradation of Numb, implicated in the control of cell fate decisions during development. DIPA interacts with the viral phosphoprotein hepatitis delta antigen (HDAG) and acts as a repressor of gene transcription [21]. SPG21 binds to the hydrophobic C-terminal amino acids of CD4 which are involved in repression of T cell activation [22]. In *D. melanogaster*, the subolesin ortholog *bhringi *(*bhr*; CG8580) may act to regulate Twist activity through recruitment of the chromatin remodeling Brahma complex [17]. Therefore, subolesin may exert its effect on gene expression through the interaction with GI, GII and possibly other regulatory proteins. Interestingly, the GII knockdown phenotype was similar to that obtained with subolesin, suggesting that these proteins may functionally interact in ticks.

The results of the microarray analysis of gene expression profile in ticks after subolesin knockdown provided evidence for the role for subolesin in gene expression. Subolesin knockdown affected the expression of genes involved in multiple cellular pathways. The nonspecific effect of dsRNA injection on tick global gene expression was not addressed in these studies. However, the injection of an unrelated dsRNA did not affect the expression of selected genes differentially expressed after subolesin knockdown. Although we cannot rule out off-target effects of subolesin RNAi in ticks, evidence suggested that this was not a likely possibility to explain the effect of subolesin knockdown on tick gene expression pattern. Firstly, we did not find complementary sequences between subolesin and identified differentially expressed genes that could support off-target effects of subolesin RNAi. To search for complementary sequences between subolesin and identified differentially expressed genes, we used the approach proposed by Birmingham et al. [23] who showed that although maximum complementarity by itself is an unsatisfactory predictor of off-target RNAi effects, a highly significant association exists between off-targeting and exact complementarity between the seed region (bases 2–8) of siRNA and their off-targeted gene 3' untranslated region (UTR). Secondly, the analysis of *D. melanogaster *subolesin ortholog RNAi off-target effects demonstrated the presence of a single off-targeted gene [24], suggesting that off-target effects of subolesin RNAi may also be minimal in ticks.

A common characteristic of many regulatory protein sequences is the presence of phosphorylation sites. Although we did not demonstrate phosphorylation of tick subolesin, there is evidence that the human ortholog protein undergoes phosphorylation at serine 21 (AS*PKRRR) [25], a PKC phosphorylation site that is conserved in tick sequences. Therefore, as with other regulatory proteins, subolesin may be regulated by reversible phosphorylation by PKC.

## Conclusion

**Figure 8:**
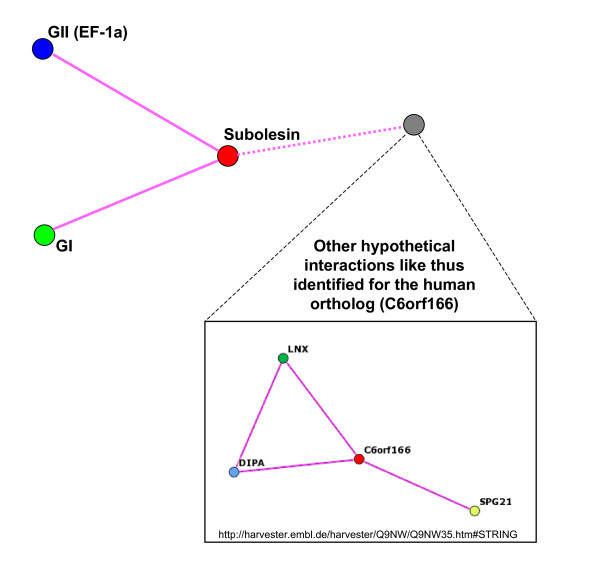
**The regulatory function of subolesin on gene expression may be exerted through interactions with regulatory proteins that act at the transcriptional and/or translational levels.** Tick subolesin interacts with GI and GII proteins with putative regulatory functions on gene expression. Other hypothetical interactions with regulatory proteins may exist based on data for the human ortholog. The search for human ortholog (C6orf166; [Genbank:NP_060534]) protein-protein interactions was done by STRING.

In summary, the results presented herein provide evidence that support a role for subolesin in gene expression in ticks and other organisms. The regulatory function of subolesin in gene expression may be exerted through interaction with other regulatory proteins at the transcriptional level (Fig. 8). In fact, a recent publication by Goto et al. [26] that appeared after submission of our work renamed subolesin orthologs in insects and vertebrates as Akirins and proposed that they constitute transcription factors required for NF-kB-dependent gene expression in *Drosophila *and mice. Alternatively, subolesin may also interact with proteins involved in translational control in ticks (Fig. 8). The experimental approach used in this study may be important for the annotation of tick sequences that result from genome sequencing efforts [27] and for the characterization of candidate tick protective antigens for the development of vaccines for the control of tick infestations and the transmission of tick-borne pathogens [3].

## Methods

### Ticks

*I. scapularis *female ticks were obtained from the laboratory colony maintained at the Oklahoma State University tick rearing facility. Off-host ticks were maintained in a 12 hr light: 12 hr dark photoperiod at 22–25°C and 95% relative humidity. *R. microplus *female ticks (Mozambique strain) were reared in Holstein-Friesian cattle at the Utrecht Centre for Tick-borne Diseases, Utrecht University. Animals were housed with the approval and supervision of the respective Institutional Animal Care and Use Committees.

### cDNA library construction

*R. microplus *eggs were used for RNA extraction and cDNA library construction. cDNA synthesis and amplification was performed using the Super SMART System™ principle (Clontech Laboratories, Mountain View, CA, USA) with adapted anchor primers containing unique *Sfi*I restriction sites for directional cloning (SMART IV: 5'-AAGCAGTGGTATCAACGCAGAGTGGCCATGGAGGCCGGG-3'and CDS III: 5'-ATTCTAGAGGCCTCCATGGCCGACATG(T)30VN-3'). Amplified cDNA was polished using the protocol described by the SMART PCR cDNA synthesis manual (Clontech Laboratories), purified using the DNA Extract II Kit (Macherey-Nagel, Düren, Germany) and subjected to directional cloning in pACT2 plasmid via the *Sfi*I sites. Repetitive electroporation of *E. coli *JM109 cells was performed to obtain a library with a titer exceeding 3 × 10^7 ^cfu/ml.

### Yeast two-hybrid screen

The yeast two-hybrid screen was performed as dictated in the MATCHMAKER Two-Hybrid user manual (Clontech Laboratories). Full-length subolesin cDNA from *R. microplus *was inserted in yeast expression vector pAS2_1 at *Nde*I and *Pst*I sites. Yeast strain AH109 was co-transformed with plasmid pAS2_1_subolesin (bait) and pACT2 *R. microplus *egg cDNA library (prey) in sequential transformation procedure. The co-transformants were selected after incubation at 30°C for 5 days on synthetic defined (SD) minimal medium lacking tryptophan and leucine. All colonies were pooled from the plates and replated on SD minimal medium lacking tryptophan, leucine, histidine and adenine. Positive clones were analyzed for β-galactosidase activity by colony-filter assays. Prey plasmids of positive clones were rescued using *E. coli *KC8 and subjected to DNA sequencing.

### Confirmation of protein-protein interaction by co-affinity purification

The GI and GII cDNA fragments, encoding for putative prey proteins interacting with the subolesin bait, were amplified by PCR using oligonucleotide primers QEGI5: 5'-GGCCATGGAGGCCGGGATAGGA and QEGI3: 5'-GGAGATCTATGCTCATTAAGACAAATCTC, and QEGII5: 5'-GGCCATGGCCTCAACCAGGCCCACGGACAAACCCCTC and QEGII3: 5'-GGAGATCTGGCGACCGTTTGCCTCATGTC, respectively and the Access RT-PCR system (Promega, Madison, WI, USA). The PCR primers introduced *Nco*I and *Bgl*II restriction sites for cloning into the expression vector pQE-60 fused to a 6xHis tag (Qiagen Inc., Valencia, CA, USA). The full-length cDNA of *I. scapularis *subolesin was inserted into the expression plasmid pFLAG-CTC (Sigma, St. Louis, MO, USA) as described previously [8]. Recombinant subolesin, GI and GII proteins were expressed in *E. coli *JM109 as described previously [8]. For co-affinity purification of subolesin-interacting proteins, the Ni-NTA spin columns (Qiagen) were used following manufacturer's protocol. The supernatant of GI and GII cell lysates were loaded onto the columns. The columns were washed three times with washing buffer (Qiagen) before loading the purified subolesin. The columns were incubated at 4°C for 4 hours to allow protein-protein interaction and then washed again. The proteins were eluted with elution buffer (Qiagen) and the eluted proteins were subjected to 12% sodium dodecyl sulfate-polyacrylamide electrophoresis (SDS-PAGE) and immunoblotted with anti-subolesin antibodies [8]. Controls included affinity purification of GI and GII in the absence of subolesin and loading subolesin onto Ni-NTA columns in the absence of His-tagged GI and GII proteins and in the presence of an unrelated His-tagged protein. GI and GII proteins were detected by Western blot using the HisDetector Western Blot Kit HRP (KPL, Gaithersburg, Maryland, USA).

### RNA interference in ticks

Oligonucleotide primers homologous to *I. scapularis *and *R. microplus *subolesin containing T7 promoters were used for *in vitro *transcription and synthesis of subolesin dsRNA as described previously [10,12], using the Access RT-PCR system (Promega) and the Megascript RNAi kit (Ambion, Austin, TX, USA). For the synthesis of *R. microplus *GI and GII dsRNAs, oligonucleotide primers GI5: 5'-ATGGAGGCCGGGATAGGA and GI3: 5'-TGCTCATTAAGACAAATCTC, and GII5: 5'-GGCCCACGGACAAACCCCTC and GII3: 5'-CGACCGTTTGCCTCATGTC were used, respectively, with the same procedure described above. The dsRNA was purified and quantified by spectrophotometry.

Unfed *I. scapularis *female ticks were injected with approximately 0.5 μl dsRNA in the lower right quadrant of the ventral surface of the exoskeleton of ticks [10]. Freshly molted *R. microplus *females were placed on double-sided sticky tape with the ventral side upwards and injected with approximately 0.5 μl dsRNA into the anal aperture using a syringe mounted on a MM3301-M3 micromanipulator (World Precision Instruments (WPI), Berlin, Germany) and connected to an UMPII syringe pump (WPI) [12]. Engorged *R. microplus *females were injected with 5 μl of dsRNA (5 × 10^10^–5 × 10^11 ^molecules/μl) in the right spiracular plate within 6 hours after dropping off the host [12]. The injections were done using a 10 μl syringe with a 1 inch, 33 gauge needle (Hamilton, Bonaduz, Switzerland). Control ticks were injected with injection buffer (10 mM Tris-HCl, pH 7, 1 mM EDTA) alone (saline negative control). We demonstrated previously that there is no difference between using an unrelated dsRNA or injection buffer alone for negative control in tick RNAi experiments [10].

Two RNAi experiments were conducted with *I. scapularis*. In the first experiment, designed to determine the time after dsRNA injection in which subolesin knockdown occurs, 20 female ticks per group were injected with subolesin dsRNA or injection buffer. The ticks were held in a humidity chamber for 1 day after which they were allowed to feed on a sheep for 10 days. Three ticks from each group were collected at 1, 3, 6, 9 and 11 days post injection (dpi). Only the ticks collected after 10 days of feeding (11 dpi) were fed together with male ticks to evaluate the effect of subolesin knockdown on tick weight. After collection, ticks were weighed and the tick weight was compared between subolesin dsRNA-injected and saline controls by Student's t-test (P = 0.05). Internal organs were then dissected and stored in RNALater (Ambion) for RNA extraction to determine subolesin mRNA levels by real-time RT-PCR. In the second experiment, 100 *I. scapularis *female ticks per group were injected with subolesin dsRNA or injection buffer alone. The ticks were held in a humidity chamber for 1 day and then allowed to feed on a sheep without males for 5 and 8 days (6 and 9 dpi) in groups of 50 dsRNA- and 50 saline-injected ticks each. Ten additional female ticks per group were fed with males for 10 days to compare the weight of replete dsRNA-injected and control ticks after RNAi by Student's t-test with unequal variance (P = 0.05). Unattached ticks were removed two days after infestation. After feeding, ticks were removed from the sheep and dissected for RNA extraction.

For RNAi in *R. microplus*, unfed and replete female ticks were injected with GI dsRNA, GII or subolesin dsRNA or injection buffer alone as described previously [12]. In the experiment with unfed ticks, 60–76 freshly molted female ticks were injected and fed on a calf for 15 days with untreated males to determine female tick mortality, weight, oviposition and hatching. Tick mortality was evaluated as the ratio of dead female ticks 15 dpi to the total number of female ticks placed on the calf and was compared between dsRNA and mock-injected control ticks by χ^2^-test as implemented in Mstat 4.01 (α = 0.01). Ticks completing feeding and egg masses oviposited by each tick were weighed individually and the average ± SD were calculated and compared between dsRNA and saline injected control ticks by Student's t-test with unequal variance (P = 0.05). In the experiment with replete female ticks, 6 ticks were used per group. Each tick was weighed before injection and stored individually in 2 ml Eppendorf tubes with pierced lids in an incubator at 27°C and 95% relative humidity to evaluate oviposition and hatching after RNAi. The weight of replete ticks before injection and the eggs mass per tick were compared with the control saline injected ticks by Student's t-test with unequal variance (P = 0.05). mRNA levels of RNAi targeted genes were determined by real-time RT-PCR. For real-time RT-PCR, internal organs were dissected and total RNA was extracted from 6 females of each dsRNA- or saline-injected group after 6 days of feeding and from 100 mg eggs oviposited by injected replete ticks.

### Suppression-subtractive hybridization (SSH)

Total RNA was isolated from pooled guts and salivary glands of *I. scapularis *female ticks injected with subolesin dsRNA or injection buffer at 9 dpi (8 days of feeding) using TriReagent (Sigma) according to manufacturer's instructions. RNA quality was assessed by gel electrophoresis. SSH was performed at Evrogen JCS (Moscow, Russia) as described previously [6,28]. Tester and driver RNAs were subtracted in both directions to construct two SSH libraries enriched for differentially expressed cDNAs in subolesin dsRNA-injected (reverse-subtracted) and saline-injected (forward-subtracted) ticks. Approximately 100 clones from each library were randomly picked up and subjected to differential hybridization with subtracted and non-subtracted probes using the PCR-select differential screening kit (Clontech, Palo Alto, CA, USA), which resulted in > 95% candidate differentially expressed cDNAs.

### Microarray construction and analysis

Tick cDNA fragments (384 from each of the reverse and forward subtracted SSH libraries) were amplified by PCR using pAL-16 vector-specific primers, purified (MultiScreen PCR plates, Millipore, Billerica, MA, USA) and arrayed onto gamma amino propyl silane coated GAPS II slides (Corning, Lowell, MA, USA). Eight pools of 12 clones each from an unsubtracted *I. scapularis *cDNA library [28] and subolesin cDNA were also arrayed and used to validate normalization. Total RNA was prepared from subolesin dsRNA- and saline-injected ticks at 6 and 9 dpi (5 and 8 days of feeding) using the RNeasy Mini Kit (Qiagen) including the on-column DNA digestion with the RNase-free DNase set following manufacturer's instructions. Total RNA (5 μg) were labeled using the 3DNA Array900 kit with Alexa Fluor dyes (Genisphere, Hatfield, PA, USA), Superscript II (Invitrogen, Carlsbad, CA, USA), the supplied formamide-based hybridization buffer and 24 × 60 mm LifterSlips (Erie Scientific, Portmouth, NH, USA) according to the manufacturer's (Genisphere) instructions. Hybridization signals were measured using a ScanArray Express (PerkinElmer, Boston, MA, USA) and the images were processed using GenePix Pro version 4.0 (Axon, Union City, CA, USA). Ratios were calculated as subolesin dsRNA-injected ticks versus saline-injected control ticks. Normalized ratio values obtained for each probe were averaged across 50 biological replicates and four technical replicates and significant differences were defined as displaying an expression fold change greater than 2-fold. All the microarray data were deposited at the NCBI Gene Expression Omnibus (GEO) under the platform accession number GPL6394 and the series number GSE10222.

### Real-time RT-PCR analysis

The RNA samples prepared as described above from *I. scapularis *female ticks and from *R. microplus *female ticks and eggs after RNAi experiments were used for real-time RT-PCR analysis. The RNA from *I. scapularis *female ticks injected with an unrelated 4A8 dsRNA [29] was used as a control in the analysis of mRNA levels for selected differentially expressed genes after subolesin RNAi. Two primers were synthesized based on the sequences determined for subolesin, GI, GII and three differentially expressed genes after subolesin RNAi (identical to *I. scapularis *putative secreted salivary WC peptide [Genbank:AAY66498] and putative secreted protein [Genbank:AAM93633] and *M. rosenbergii *Cu-Zn SOD; [Genbank:AAZ29240]) and used for real-time RT-PCR analysis of mRNA levels in dsRNA- and control ticks. Real-time RT-PCR was done using the QuantiTec SYBR Green RT-PCR kit (Qiagen, Valencia, CA, USA) and a Bio-Rad iQ5 thermal cycler (Hercules, CA, USA) following manufacturer's recommendations and oligonucleotide primers and PCR conditions described in Table 4. mRNA levels were normalized against tick β-actin using the comparative Ct method. mRNA levels were compared between dsRNA-infected and control ticks by Student's t-Test (P = 0.05).

**Table 4:** RT-PCR oligonucleotide primers and conditions.

**Gene description**	**Upstream/downstream primer sequences (5'-3')**	**PCR annealing conditions**
***R. microplus***		

GI [Genbank:EU436162]	CACCATCACTGAAAGCGGT GTGTTAAATAGTTATGCTCATTAAGAC	50°C, 30 sec
GII [Genbank:EU436163]	GATCATTGACCTGGTACCTTCC GACTTGATGACACCGACGG	50°C, 30 sec
Subolesin [Genbank:DQ923495]	CACAGTCCGAGTGGCAGAT GATGCACTGGTGACGAGAGA	50°C, 30 sec
Beta actin [Genbank:AY255624]	CAC GGT ATC GTC ACC AAC TG TGA TCT GCG TCA TCT TCT CG	50°C, 30 sec

***I. scapularis***		

Putative secreted salivary WC peptide [Genbank:AAY66498]	GATATTGATCCAGCCGGAGA ATGTCGTCCCTCCATTGTGT	60°C, 30 sec
Putative secreted protein [Genbank:AAM93633]	TGAAGGCAACCATTGCAGTT ATTGATGGCAATCCTGTGGA	56°C, 30 sec
Identical to *M. rosenbergii *Cu-Zn SOD [Genbank:AAZ29240]	TGACCTGGGCAACGTTGA ATGACGCAGCAGGCAATG	54°C, 30 sec
Subolesin [Genbank:AY652654]	AGCAGCTCTGCTTCTCGTCT TCGTACTCGTCGCGTATCTG	54°C, 30 sec
Beta actin [Genbank:AF426178]	GAGAAGATGACCCAGATCA GTTGCCGATGGTGATCACC	50°C, 30 sec

### Sequence analysis and database search

The sequences of positive clones in the yeast two-hybrid screen were sequenced and searched for homology with the PSI-BLAST algorithm through NCBI web site. Proteins with similar domain architectures were characterized with CDART [30,31].

The tick cDNAs identified in the microarray analysis as down or up-regulated by more than two-fold after subolesin RNAi were sequenced and analyzed. Multiple sequence alignment were performed to exclude vector sequences and to identify redundant (not unique) sequences using the program AlignX (Vector NTI Suite V 8.0, InforMax, Invitrogen, Carisbad, CA, USA) with an engine based on the Clustal W algorithm. Searches for sequence similarity were performed with the BLASTX program against the non-redundant (nr) peptide sequence database and the database of tick specific sequences at NCBI and VectorBase. Protein ontology was determined using the protein reference database [32]. The possibility of subolesin RNAi off-target effects were analyzed by searching for exact complementarity between the seed region (bases 2–8) of all possible 20–22 bp subolesin siRNAs and cDNA sequences of tick differentially regulated genes identified in the microarray analysis, including the sequence of 3' UTRs when known [23].

The pattern search and predictions for tick GI, GII and subolesin post-translational modifications were done by PIR searching against the PROSITE database [33]. The search for human ortholog (C6orf166; Genbank accession number NP_060534) protein-protein interactions was done by STRING [34,35].

### Nucleotide sequence accession numbers

The nucleotide sequences of GI, GII and the ESTs reported in this paper have been deposited in the GenBank database under accession numbers [Genbank:EU436162, Genbank:EU436163 and Genbank:FD482610–FD482874], respectively.

## Authors' contributions

JdlF conceived and coordinated the study, participated in its design and helped to draft the manuscript. CM-O carried out two hybrid screening. VN, PA, AMN, CA, JMPdlL, RCG, EFB and KMK carried out RNAi, microarray analysis and real-time RT-PCR experiments. MC carried out confirmation of protein-protein interactions. PA and JdlF carried out computational analyses. KMK, FJ, CG, PA and CM-O participated in study design and helped to draft the manuscript. All authors read and approved the final manuscript.
